# Potential Association Between Changes in Microbiota Level and Lung Diseases: A Meta-Analysis

**DOI:** 10.3389/fmed.2021.723635

**Published:** 2022-01-14

**Authors:** Lan Chai, Qi Wang, Caijuan Si, Wenyan Gao, Lun Zhang

**Affiliations:** ^1^Department of Rheumatology and Immunology Department, Zhejiang Hospital, Hangzhou, China; ^2^College of Pharmacy, Harbin Medical University-Daqing, Daqing, China; ^3^Department of Nutrition, Zhejiang Hospital, Hangzhou, China; ^4^Key Laboratory of Neuropsychiatric Drug Research of Zhejiang Province, Institute of Materia Medica, Zhejiang Academy of Medical Sciences and Hangzhou Medical College, Hangzhou, China

**Keywords:** lung microbiota, COPD, asthma, meta-analysis, IPF—idiopathic pulmonary fibrosis

## Abstract

**Objective:**

Lung microbiota is increasingly implicated in multiple types of respiratory diseases. However, no study has drawn a consistent conclusion regarding the relationship between changes in the microbial community and lung diseases. This study verifies the association between microbiota level and lung diseases by performing a meta-analysis.

**Methods:**

Literature databases, including PubMed, ISI Web of Science, Embase, Google Scholar, PMC, and CNKI, were used to collect related articles published before March 20, 2021. The standard mean deviation (SMD) and related 95% confidence intervals (CIs) were calculated using a random-effects model. Subgroup, sensitivity, and publication bias analyses were also conducted.

**Results:**

Six studies, comprising 695 patients with lung diseases and 176 healthy individuals, were included in this meta-analysis. The results indicated that the microbiota level was higher in patients with lung diseases than in healthy individuals (SMD = 0.39, 95% CI = 0.22–0.55, *I*^2^ = 91.5%, *P* < 0.01). Subgroup analysis based on country demonstrated that the microbiota level was significantly higher in Chinese (SMD = 1.90, 95% CI = 0.87–2.93, *I*^2^ = 62.3%, *P* < 0.01) and Korean (SMD = 0.24, 95% CI = 0.13–0.35, *I*^2^ = 78.7%, *P* < 0.01) patients with lung diseases. The microbiota level of patients with idiopathic pulmonary fibrosis (IPF) (SMD = 1.40, 95% CI = 0.42–2.38, *I*^2^ = 97.3%, *P* = 0.005), chronic obstructive pulmonary disease (COPD) (SMD = 0.30, 95% CI = 0.09–0.50, *I*^2^ = 83.9%, *P* = 0.004), and asthma (SMD = 0.19, 95% CI = 0.06–0.32, *I*^2^ = 69.4%, *P* = 0.004) were significantly higher than those of the healthy group, whereas a lower microbiota level was found in patients with chronic hypersensitivity pneumonitis (CHP). The microbiota level significantly increased when the disease sample size was >50. Subgroup analysis based on different microbiota genera, indicated that *Acinetobacter baumannii* and *Pseudomonas aeruginosa* were significantly increased in COPD and asthma diseases.

**Conclusion:**

We observed that patients with IPF, COPD, and asthma had a higher microbiota level, whereas patients with CHP had a lower microbiota level compared to the healthy individuals. The level of *A. baumannii* and *P. aeruginosa* were significantly higher in patients with COPD and asthma, and thus represented as potential microbiota markers in the diagnosis and treatment of lung diseases.

## Introduction

An abundance of microbiota lives both inside and outside the human body. Investigation of the microbiota has contributed dramatically to our understanding of their critical role in multiple human diseases, such as cancers, infectious diseases, and lung diseases (1–4). The “Human Microbiome Project” was launched in 2007 to understand the complexity of the human microbiome, which has also contributed to the understanding of pathogenesis in a wide range of human diseases (5). The human microbiota comprises bacteria, archaea, viruses, and eukaryotes. These microbiota communities affect human physiological functions, both in health and disease status, contributing to the enhancement or impairment of metabolic and immune functions (6). Alterations in the microbiota level and composition may lead to an ecological imbalance by decreasing the number of symbionts and increasing potentially dangerous pathogens (7).

Lungs are vital organs for gaseous exchange during respiration. For many years, the lungs have been thought to be a sterile environment (8). With advancements in molecular and biochemical techniques, many studies have reported that microbial imbalance, known as dysbiosis, contributes to the occurrence, development, and deterioration of lung diseases (9). A previous meta-analysis identified several taxa annotated for *Rummeliibacillus* sp. species, *Deinococcus, Kurthia, Brevibacillus borstelensis, Caulobacter* sp., *Actinomyces graevenitzii, Rhodotorula mucilaginosa*, and *Mycobacterium tuberculosis*, which were significantly abundant in tuberculosis (TB) cases. Other taxa, including *Clostridiales, Tumebacillus ginsengisoli, Pelomonas aquatica, Propionibacterium acnes*, and *Haemophilus parahaemolyticus*, were lower in patients with TB than in healthy individuals (10). However, since 2010, studies have also described seeing alterations of microbiota in other lung diseases, such as chronic obstructive pulmonary disease (COPD), idiopathic pulmonary fibrosis (IPF), and asthma indicating that the lung microbiota influenced both healthy and diseased subjects (11–13). COPD is the third leading cause of death worldwide (14). Whereas, IPF is a progressive lung disease that can lead to rapid death, and has an average survival rate of 2–3 years following diagnosis, mainly in older adults (15, 16). Although some studies had demonstrated a significant association between the lung microbiota and IPF, the key questions remained unanswered. Asthma kills ~1,000 people every day and affects over 330 million people worldwide (17). In addition, there is a growing interest in investigating microbiome interactions and identifying specific microbes or microbial products as potential new treatment targets (18).

Recently, pyrosequencing of the 16S ribosomal RNA (rRNA) gene amplicons from bronchoalveolar lavage specimens, bronchial brushings, and lung tissues from patients with lung diseases and healthy subjects demonstrated that various microorganisms were abnormally altered at the molecular level (19–23). However, assessment of the lung microbiota in the pathogenesis of these diseases has not yet been wholly and systematically conducted. Moreover, it is difficult to characterize the bacterial community in multiple lung diseases using conventional culture methods. There is also no scientific evidence or a clear consensus regarding this association from previous studies. Meta-analysis has the advantage of reducing errors by pooling a large amount of available data and providing more precise estimates. The purpose of this meta-analysis was to verify the association between microbiota level and lung diseases. Studies were included based on the inclusion and exclusion criteria, and data were extracted from these studies. Standard mean deviation (SMD) and related 95% confidence intervals (CIs) were calculated followed by sensitivity, publication bias assessment, and subgroup analyses.

## Materials and Methods

This meta-analysis was guided by the Preferred Reporting Items for Systematic Reviews and Meta-Analysis (PRISMA) guidelines (24).

### Search Strategy

Two investigators independently performed a literature search using PubMed, ISI Web of Science, Embase, Google Scholar, PMC, and Chinese National Knowledge Infrastructure (CNKI), which was published before March 20, 2021. The publication language was limited to English and Chinese. Each database used a different search strategy. For example, in PubMed, the following MeSH terms and keywords were used: (“microbiota”[MeSH Terms] OR “microbiome”[MeSH Terms] OR “microbiota”[text word] OR “microbiome”[text word] OR “bacteria”[MeSH Terms] OR “bacteria”[textword]) AND (“lung diseases”[MeSH Terms] OR “lung diseases”[text word] OR “asthma”[MeSH Terms] OR “COPD”[MeSH Terms] OR “pneumonia”[MeSH Terms]). In addition, all irrelevant studies, such as case reports, comments, and review articles, were excluded. Relevant studies cited in review articles were also manually searched and included as eligible studies.

### Inclusion and Exclusion Criteria

Two investigators independently read the titles and abstracts of the searched articles to determine whether they met the inclusion criteria. Both cross-sectional and longitudinal studies on the relationship between microbiota level and lung diseases were included. Studies were included in this meta-analysis if they met the following criteria: (1) included major types of lung diseases, such as COPD, asthma, pulmonary fibrosis, or pneumonitis; (2) the diagnosis of these diseases was clinically confirmed according to the disease guidelines; (3) the study included healthy individuals as a control group; (4) microbiota level was detected in subjects with lung diseases and the control group; (5) the number of case samples and control groups was provided; and (6) the study was published in Chinese or English. Articles were excluded if they: (1) were case reports, comments, animal or cell articles, or review articles; (2) duplicate articles; (3) insufficient data to allow for the extraction of microbiota expression level in patients and controls; and (4) articles not related to microbiota, microbiome, or bacteria.

### Data Extraction and Quality Assessment

Two investigators independently performed data extraction, which included the following information from each study: first author, year of publication, age of cases, ethnicity, the number of patients and controls, patient characteristics, sample type, microbiota detection methods, mean and standard deviation (SD) or standard error (SE) values of microbiota in both patients and control individuals. Any inconsistencies were resolved by other researchers until a consensus was reached.

The quality assessment of the studies included in this meta-analysis was conducted using the Newcastle–Ottawa Scale (NOS) (25), which could evaluate the risk of bias of all included case–control studies. The NOS assesses three domains: selection bias, group comparability, and cohort exposure. The total NOS score ranges from 0 to 9, with higher scores indicating better quality.

### Statistical Analysis

The mean and SD values of microbiota of the patients and control groups were extracted from the included studies. The SMD and related 95% CIs as the magnitude of the effect were calculated as the amount of the combined effect. Subgroup analysis was conducted based on country, disease difference, and case sample size > 50. Heterogeneity across studies was assessed using *I*^2^ statistics. *I*^2^-values of 25, 50, and 75% were assigned as low, moderate, and high degrees of heterogeneity, respectively. The pooled effect was determined using a random effect model when the *I*^2^ value was >75%. Otherwise, a fixed-effects model was used. A sensitivity analysis was performed to evaluate the influence of each study. Begg's and Egger's tests were used to assess the potential publication bias. All statistical analyses were conducted using Stata software version 12.0 (Stata Corp LP, TX, United States). Statistical significance was set at *P* < 0.05.

## Results

### Characteristics and Quality of the Included Studies and Subjects

**Figure 1:**
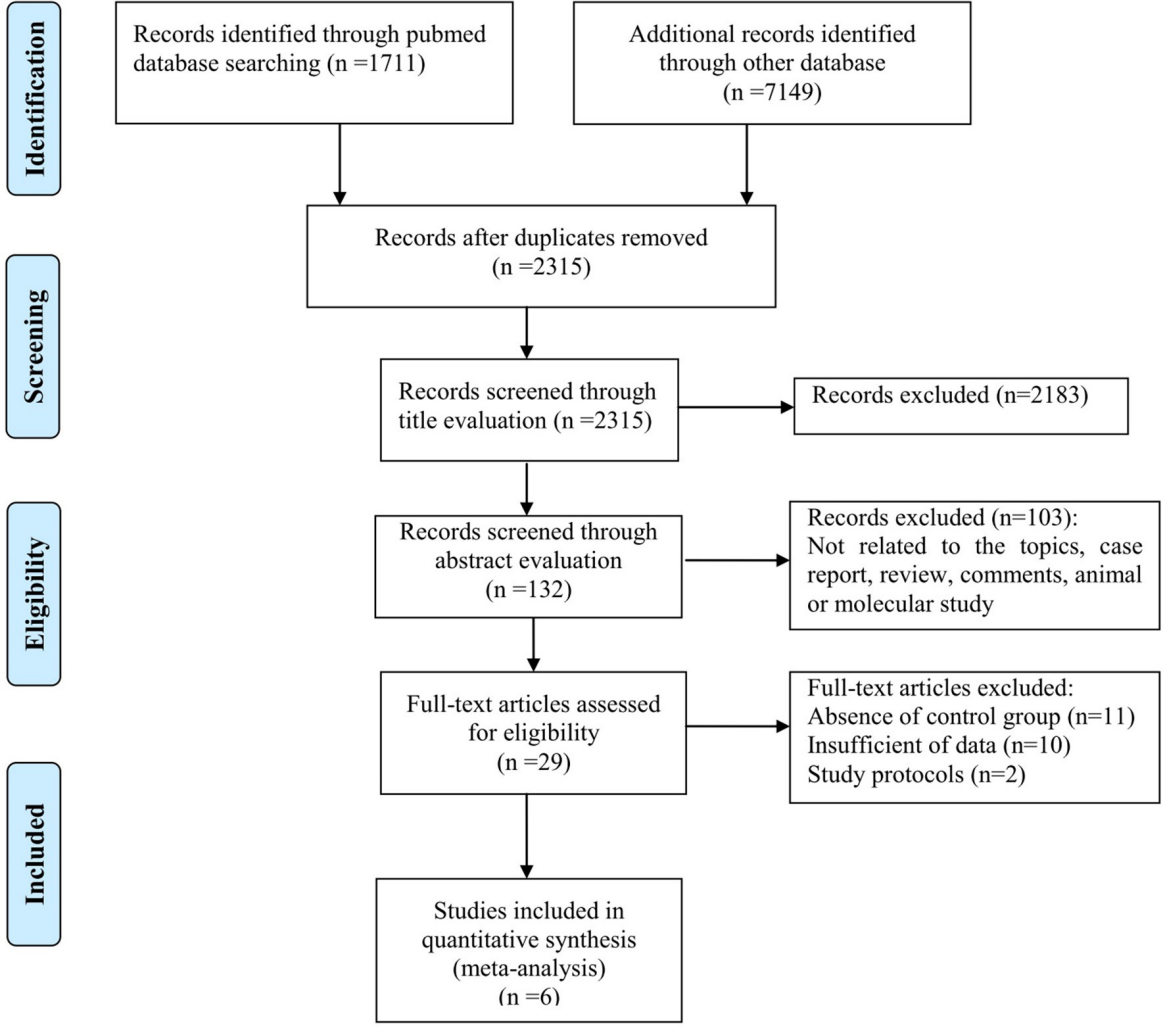
Flowchart showing the selection process for the studies.

From the literature search, a total of 8,860 articles were retrieved from PubMed, ISI Web of Science, Embase, and other databases. All studies were screened by reading their titles, and 6,545 duplicates were excluded. After reading the abstracts, 2,183 studies were excluded because they were not related to the topic. We then carefully read the full text of the remaining 132 studies. Case reports, reviews, and molecular studies were excluded. Finally, we read the full text of 29 articles that met the inclusion criteria. Studies that did not provide clinical data were excluded. Therefore, we finally obtained six articles that met the inclusion criteria. A detailed schematic of the literature search is shown in Figure 1.

The characteristics of the included studies and the subjects were presented in Table 1. The NOS quality scores of the included studies ranged from 6 to 9 as shown in Table 1. Overall, the included studies comprised 871 individuals (695 patients and 176 healthy individuals), which were published between 2014 and 2020, and conducted in the United Kingdom, United States, Korea, and China. Among these articles, three studies included individuals of Caucasian ethnicity (19, 20, 22), while three studies included individuals of Asian ethnicity (21, 23, 26). The age of the case groups was 50–68 years. The sample size varied from 18 to 532 in the included studies. Moreover, they contained multiple comparisons. Therefore, we treated each group as an independent comparison. The lung microbiota samples were sourced from serum, lung tissue, sputum, and bronchoalveolar lavage fluid samples. The 16S rRNA gene sequence method was used in all included studies for microbiome screening. DNA was extracted from the microbiota, and quantitative reverse transcription-polymerase chain reaction (qRT-PCR) was used to detect the expression level of each microbiota. The subjects had COPD, asthma, IPF, or chronic hypersensitivity pneumonitis (CHP) diseases. In addition, three studies showed an association between bacterial species, such as *Streptococcus pneumonia, Klebsiella pneumoniae*, and *Pseudomonas aeruginosa*, and COPD (19, 23, 26). Two studies showed that several kinds of bacteria were related to IPF disease, including *Haemophilus, Neisseria*, and *Streptococcus* (20, 22). Yang et al. found that several types of bacteria had a potential relationship with asthma, for example, *Acinetobacter baumannii, Enterobacter cloacae, P. aeruginosa*, and *Staphylococcus aureus* among others (26). *Prevotella, Streptococcus, Veillonella, Neisseria*, and other bacteria were related to the occurrence and development of the CHP disease (20).

**Table 1:** Characteristics of the included studies and subjects.

**First author**	**Year**	**Country**	**Study design**	**Disease**	**Study group**	**No**.	**Age of case group(year)**	**Sample source**	**Microbiota type**	**Detection content**	**Detection method**	**NOS score**
Dachang Wu	2014	China	Case-control study	COPD	COPD HC	10 10	60–80	sputum	*Streptococcus pneumonia Klebsiella pneumonia Pseudomonas aeruginosa*	DNA	PCR, 16S rRNA gene	7
Jinho Yang	2020	Korea	Case-control study	Asthma COPD	Asthma COPD HC	239 205 88	55.5 ± 14.5 66.4 ± 7 50.8 ± 9.8	serum	*S. aureu, A. baumannii, E. cloacae, P. aeruginosa*	bacterial EV immuno globulin G	16S rRNA gene, ELISA	9
Hyun Jung Kim	2017	Korea	Case-control study	COPD	COPD HC	13 13	65.5 ± 7.8 65.5 ± 7.8	lung tissue	*Firmicutes*	DNA	16S rRNA gene	7
Phillip L	2014	United Kingdom	Prospective study	IPF	IPF HC	65 27	68 ± 68.2 58.2 ± 68.0	BAL fluid	*Haemophilus* *Neisseria* *Streptococcus* *Veillonella*	DNA	16S rRNA gene	8
Rachele Invernizzi	2020	United States	Prospective study	CHP IPF	CHP IPF HC	110 45 28	66 ± 9 62 ± 19 55± 15	BAL fluid	*Prevotella* *Streptococcus* *Veillonella* *Neisseria* *Haemophilus* *Actinomyces* *Rothia* *Fusobacterium*	DNA	16S rRNA gene	9
Simon JS	2016	United Kingdom	Case-control study	COPD	COPD HC	8 10	67.75 52.9	sputum	*Fusobacterium* *Haemophilus* *influenzae* *Ochrobactrum* *anthropi* *Streptococcus* *pneumoniae* *Streptococcus* *thermophilus*	DNA	16S rRNA gene	6

*Note: COPD, chronic obstructive pulmonary disease; IPF, idiopathic pulmonary fibrosis; CHP, chronic hypersensitivity pneumonitis; BAL, broncho-alveolar lavage; HC: healthy control*.

### Meta-Analysis: The Association Between Microbiota Level and Lung Diseases

**Figure 2:**
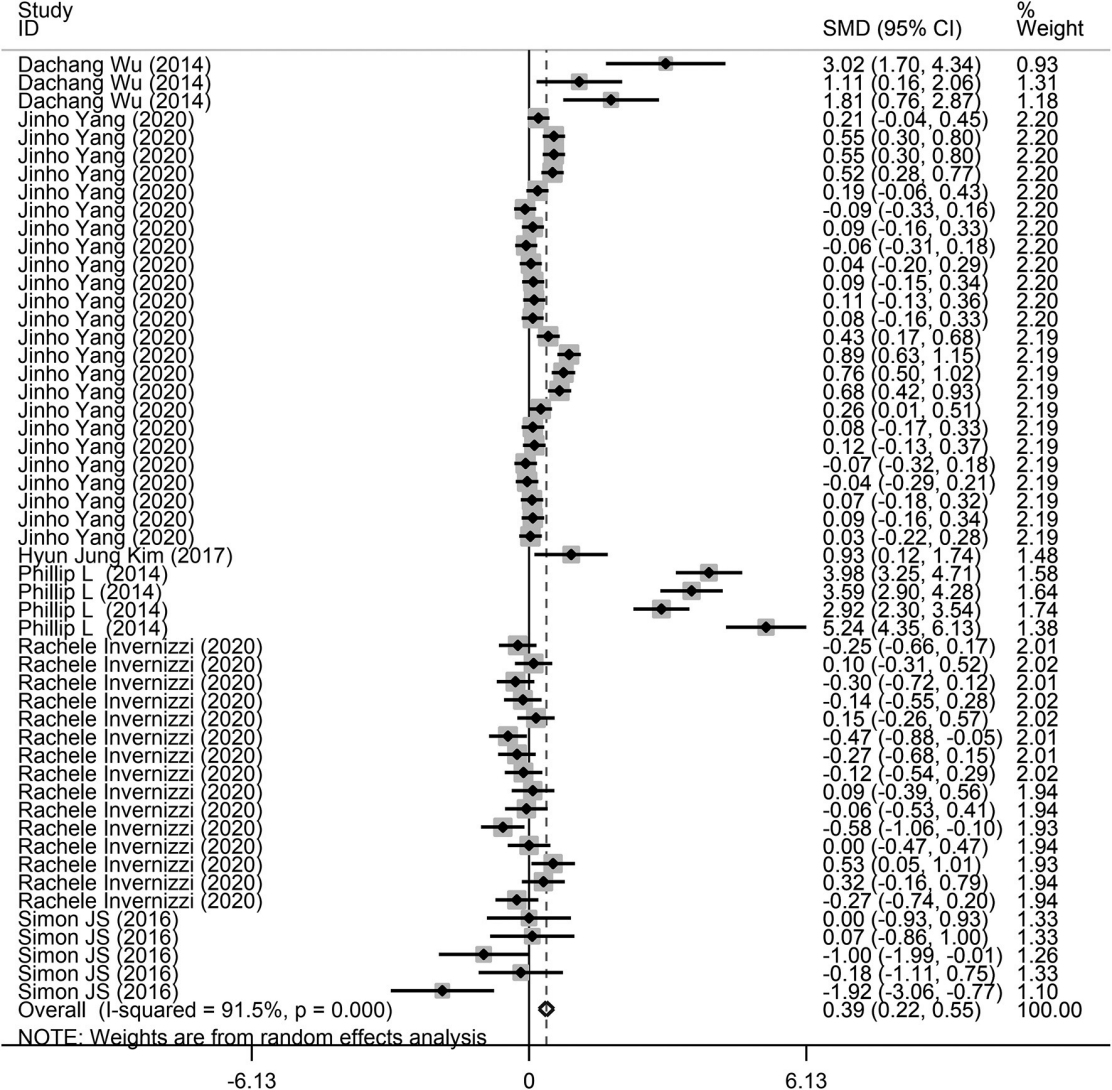
Forest plots of the association between microbiota expression level and patients with lung diseases. This is the overall analysis. For each study, the estimate of differences in mean microbiota level and its 95% confidence interval (95% CI) is plotted with a diamond. SMD, standard mean difference; Chi^2^, Chi-square statistic; df, degrees of freedom; *I*^2^, I-square heterogeneity statistic; IV, inverse variance; Z, Z-statistic.

Fifty-two comparisons were performed to evaluate the microbiota level in subjects with lung diseases and control individuals, with a total of 871 subjects. Among these studies, a higher microbiota level was observed in subjects with lung diseases (SMD = 0.39, 95% CI = 0.22–0.55, *I*^2^= 91.5%, *P* < 0.01) (Figure 2). Heterogeneity analysis was performed across the included studies using Cochran Q and *I*^2^ tests. The results indicated significant heterogeneity across the included studies.

**Figure 3:**
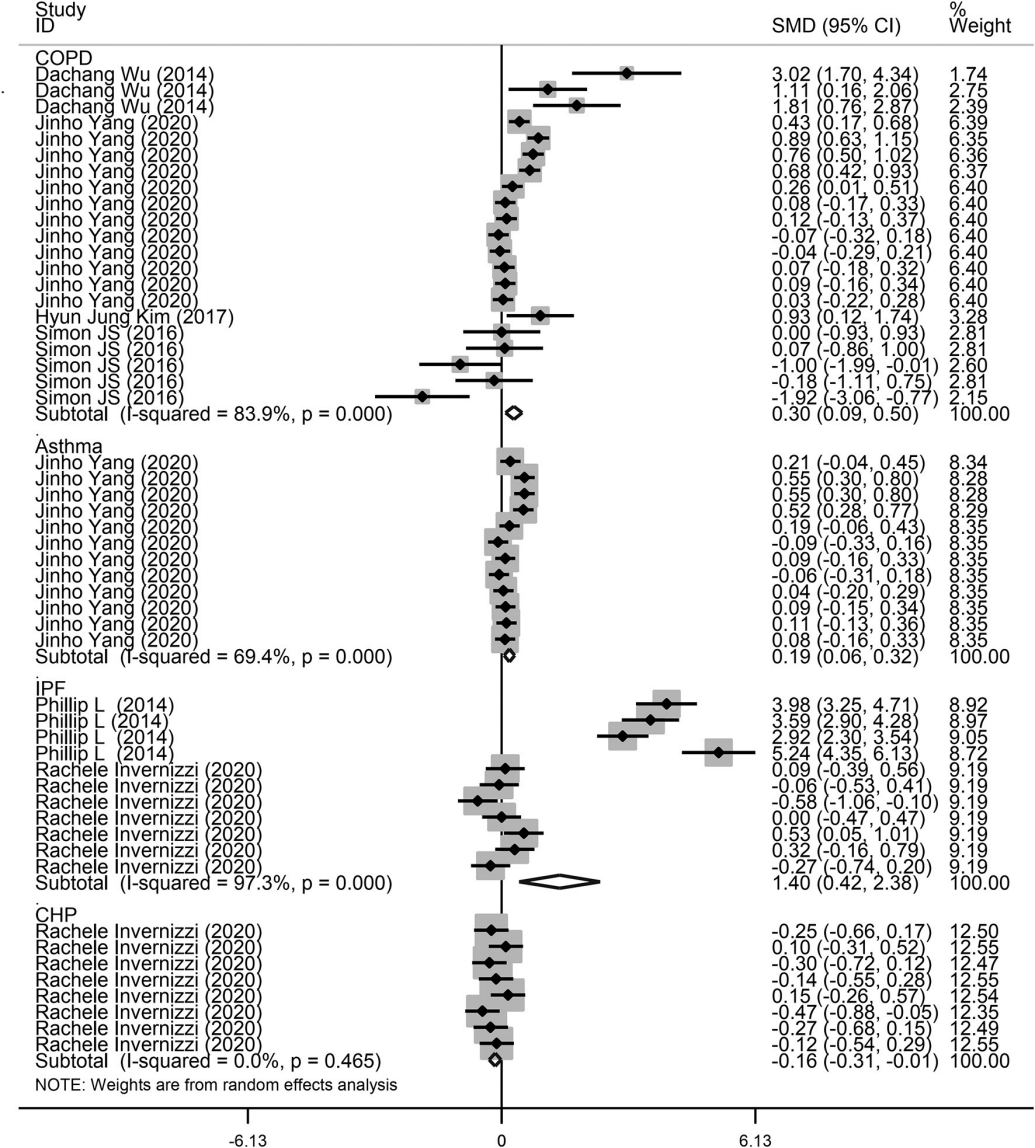
Subgroup analysis of the association between microbiota expression level and patients with lung diseases based on different countries.

**Figure 4:**
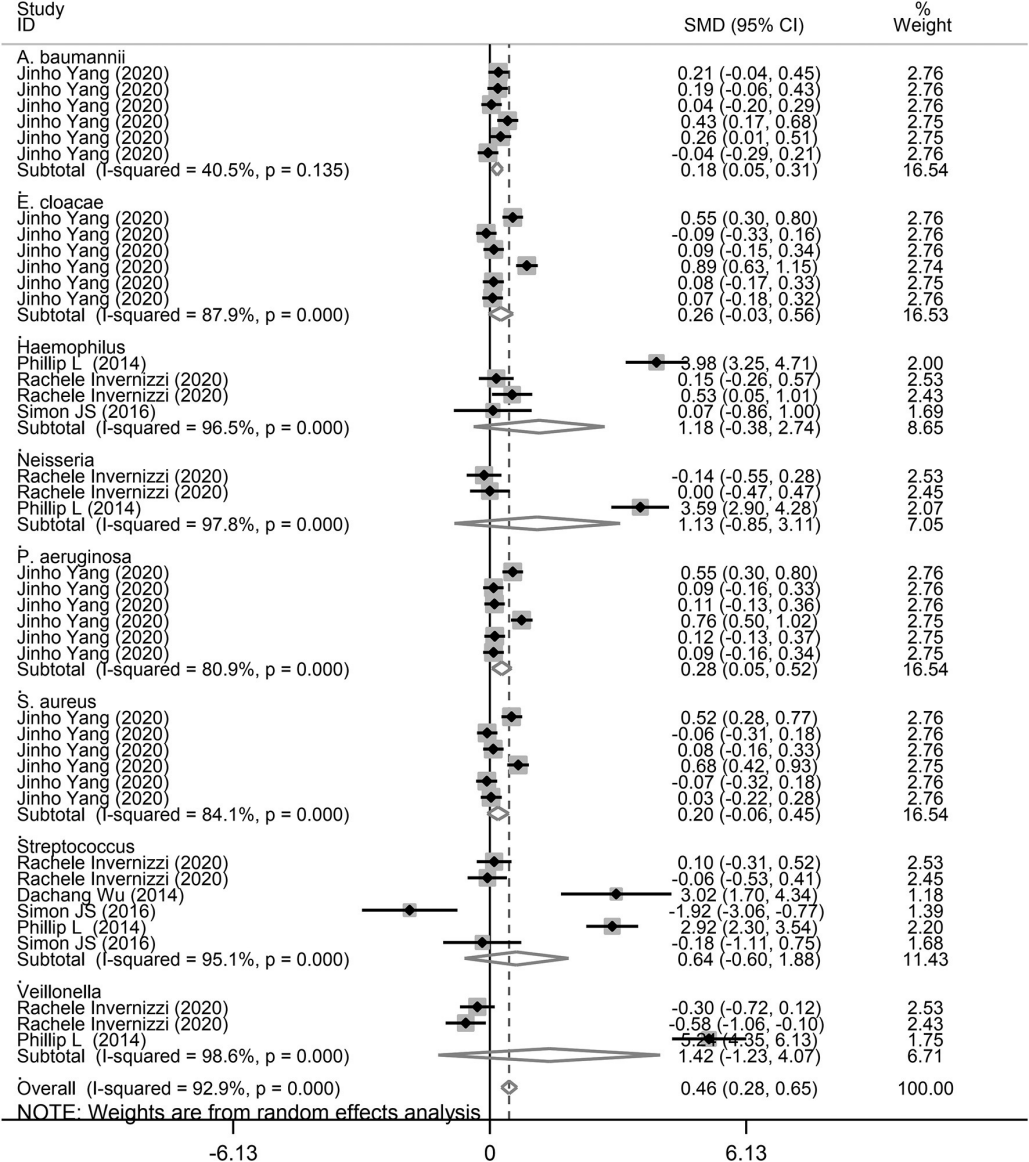
Subgroup analysis of the association between microbiota expression level and patients with lung diseases based on different lung diseases.

**Figure 5:**
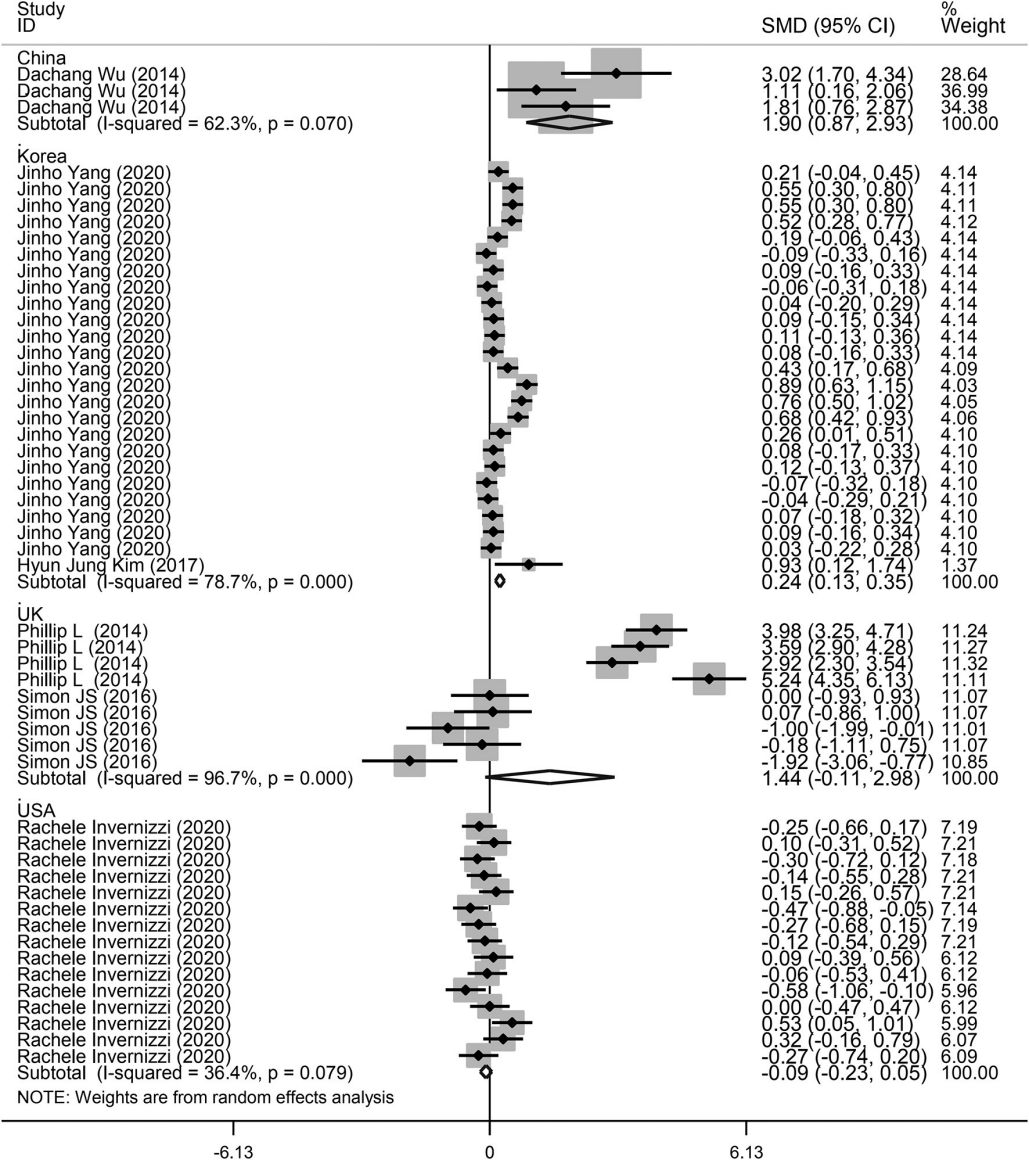
Subgroup analysis of the association between microbiota expression level and patients with lung diseases based on different sample sizes.

Next, a subgroup analysis was conducted to explore potential heterogeneity. Country, sample size, type of microbiota, and type of lung disease were used as covariates to perform subgroup analysis using Stata software. Subgroup analysis based on country demonstrated that increased microbiota levels were significantly associated with lung disease occurrence in China (SMD = 1.90, 95% CI = 0.87–2.93, *I*^2^ = 62.3%, *P* < 0.01) and Korea (SMD = 0.24, 95% CI = 0.13–0.35, *I*^2^ = 78.7%, *P* < 0.01), as shown in Figure 3. However, there was no significant association between microbiota level and lung disease in the United Kingdom (SMD = 1.44, 95% CI = −0.11 to 2.98, *I*^2^ = 96.7%, *P* = 0.068) and the United States (SMD = −0.09, 95% CI = −0.23 to 0.05, *I*^2^ = 36.4%, *P* = 0.217). Subgroup analysis based on different lung diseases revealed that IPF patients showed a significant increase in their microbiota level compared to healthy individuals (SMD = 1.40, 95% CI = 0.42–2.38, *I*^2^ = 97.3%, *P* = 0.005), which was similar to that in patients with COPD (SMD = 0.30, 95% CI = 0.09–0.50, *I*^2^ = 83.9%, *P* = 0.004) and asthma (SMD = 0.19, 95% CI = 0.06–0.32, *I*^2^ = 69.4%, *P* = 0.004), as shown in Figure 4. However, a significant decrease in the microbiota level was observed in patients with CHP disease (SMD = −0.16, 95% CI = −0.31 to −0.01, *P* = 0.033), as shown in Figure 4. The effect of sample size on microbiota level in the disease and control groups was also explored. As shown in Figure 5, the microbiota level increased when the sample size was >50 (SMD = 0.38, 95% CI = 0.21–0.55, *I*^2^ = 92.4%, *P* < 0.01). However, no significant difference was found when the sample size was < 50 (SMD = 0.041, 95% CI = −0.43 to 1.25, *I*^2^ = 84.6%, *P* = 0.342).

**Figure 6:**
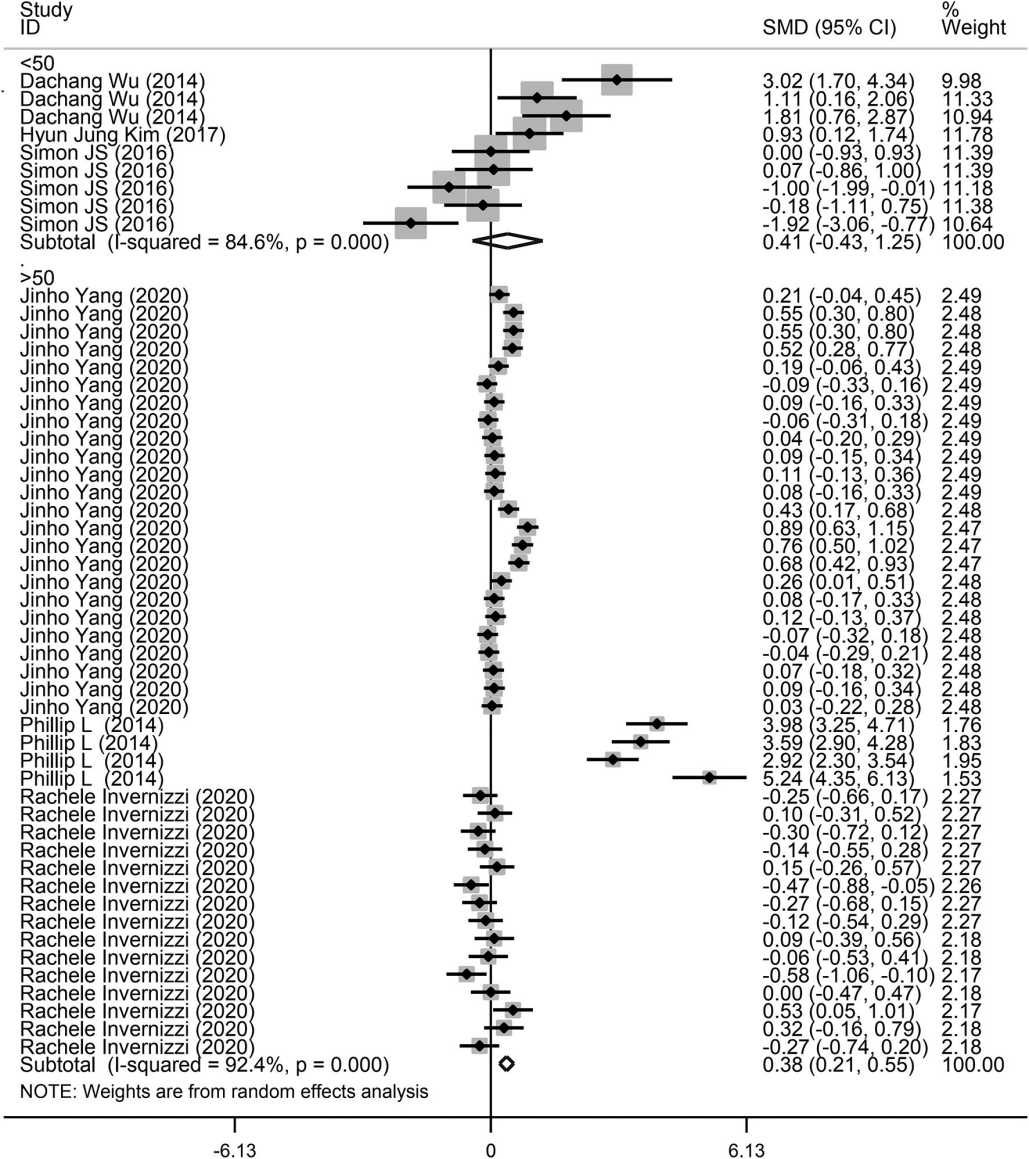
Subgroup analysis of the association between microbiota expression level and patients with lung diseases based on different microbiota genera.

Finally, we performed a subgroup analysis based on the different types of microbiota. As shown in Figure 6, *A. baumannii* (SMD = 0.18, 95% CI = 0.05–0.31, *I*^2^ = 40.5%, *P* = 0.008) and *P. aeruginosa* (SMD = 0.28, 95% CI = 0.05–0.55, *I*^2^ = 80.9%, *P* = 0.017) showed a significant increase in the study performed by Yang et al. (26), corresponding with COPD and asthma. Although there was no significant difference for *E. cloacae* in COPD and asthma, there was an increasing trend of 0.20 SMD (*P* = 0.18). Furthermore, there was no significant relationship between *E. cloacae, Haemophilus, Neisseria, S. aureus*, and *Streptococcus* genera and lung diseases. We also conducted a subgroup analysis for *A. baumannii, E. cloacae, P. aeruginosa*, and *S. aureus* in patients with asthma and COPD. As shown in Supplementary Figure 1, *A. baumannii* level was significantly increased in patients with asthma (SMD = 0.15, 95% CI = 0.004–0.286, *P* = 0.044). However, there was no significant increase in *P. aeruginosa* level (SMD = 0.25, 95% CI = −0.044 to 0.541, *P* = 0.096). In addition, there was an increasing trend for *A. baumannii* (SMD = 0.21) and *P. aeruginosa* (SMD = 0.32) levels in patients with COPD (Supplementary Figure 2).

### Sensitivity Analysis and Publication Bias

In the present meta-analysis, sensitivity analysis and publication bias were also performed using the Stata software. As shown in Supplementary Figure 3, no comparisons fell outside the lower and upper limits. Begg's and Egger's tests demonstrated that there was a low risk of publication bias in this meta-analysis (Supplementary Figure 4).

## Discussion

Numerous investigations had indicated that the lung microbiota played multiple essential roles in lung diseases (27–31). However, previous studies had limitations in their characteristics to provide strong evidence, such as small sample size and lack of compliance with modern methodological research standards. Therefore, it is difficult to draw consistent conclusions from a single trial. Thus, we performed this meta-analysis to evaluate the relationship between microbiota level and lung diseases to provide a reliable evidence-based medical investigation for clinical treatment. Here, we retrieved all publicly available lung microbiome datasets and integrated the data from six related articles, comprising 871 individuals. The present meta-analysis was the first study to indicate that patients with COPD, IPF, and asthma had a higher level of lung microbiota compared to healthy individuals. *A. baumannii* and *P. aeruginosa* were more prevalent in patients with COPD and asthma. These findings were novel, and provided insights into the potential of microbial-targeting strategies for the treatment of COPD, asthma, and other lung diseases.

Decreased microbial diversity was associated with impairment of lung function. Furthermore, the variety of lung microbiota decreased with increasing age and disease severity in lung diseases (32, 33). However, previous studies had reported that the abundance of lung microbiota was higher in lung diseases than in healthy controls (22, 23, 26). Similarly, the present meta-analysis indicated that lung microbiota was higher in lung diseases, especially in COPD, IPF, and asthma, which was consistent with previous analyses. Older adults are more likely to suffer from lung disease than individuals who are at 25–44 years of age. Moreover, the mortality risk among older patients with chronic lung disease was elevated 100-fold compared to that in healthy individuals (34). However, the effects of global environmental and geographical alterations on the lung microbiome remained poorly understood (35). For example, the differences in the ecological and air particulate exposures, lifestyle characteristics, the accuracy of microbiota sequencing, or other factors, could lead to differences in the detection of microbiome (36, 37). However, large-scale samples are needed to provide strong evidence to illustrate the role of microbiota in the pathogenesis of lung diseases.

*Acinetobacter baumannii* is a gram-negative bacterium, which was once considered harmless. Recently, *A. baumannii* had become one of the most important pathogens that poses the greatest threat to human health in clinical treatment (38). *A. baumannii* frequently causes a series of lung infections, which may lead to a high mortality rate in patients with lung disease (39). Previous researches had shown that *A. baumannii* infection induced the production of pro-inflammatory cytokines, such as TNF-α, type I IFN, and IL-1β, which mediated lung immune response and led to cell death (40–43). *P. aeruginosa* is the leading cause of a decline in lung function and has a high prevalence rate in several types of lung infections (44, 45). *P. aeruginosa* attaches to different solid surfaces and forms biofilms to enable the bacteria to resist the host's innate immune system and antibiotic treatment (46). *P. aeruginosa* is commonly found in patients with IPF and asthma (47–51). In intubated patients, *P. aeruginosa* was also found to exacerbate COPD and contribute to tissue damage (33, 52). In addition, it has five secretory systems that secrete various toxins and hydrolytic enzymes to attack the host (53, 54). It can also induce acute host inflammatory responses (55). The role of *A. baumannii* and *P. aeruginosa* in the development of lung diseases, including asthma, COPD, and IPF, remains unclear, and more tangible evidence needs to be extracted. The complexity of the lung microbiota, its genetic and metabolic properties, and manipulation as a marker for the potential treatment of lung diseases need to be studied further.

The present meta-analysis had several limitations. First, only six studies were eligible for the pooled analysis, which may have influenced the interpretation and clinical application of outcomes. Second, IPF, COPD, and asthma were simultaneously included in the meta-analysis. However, there may be a specific microbiota in each of the different lung diseases. Third, we could not perform a pooled analysis based on the various stages of the disease due to insufficient data. Finally, the detection of 16S rRNA was not continuous, therefore we could not obtain a clear consensus on the changes in the microbial community during the occurrence or progression of the disease.

## Conclusions

This meta-analysis indicated that patients with IPF, COPD, and asthma had a higher microbiota level than healthy individuals. Moreover, *A. baumannii* and *P. aeruginosa* were detected at a higher level in patients with COPD and asthma, which constituted a potential microbiota signature of the diagnosis, and treatment of these lung diseases. However, the interpretation of these results is limited by the small number of included studies and the sample size. Therefore, more studies with a rigorous methodology and larger sample sizes, including multicenter and multi-ethnic subjects, need to be performed.

## Data Availability Statement

The raw data supporting the conclusions of this article will be made available by the authors, without undue reservation.

## Author Contributions

LC, QW, and LZ designed the study and performed the meta-analysis. LC, QW, and WG wrote the draft and revised the manuscript. WG performed the literature search. CS and LZ extracted the data and processed the raw data. All authors contributed to the article and approved the submitted manuscript.

## Funding

This work was supported by the Fundamental Research Funds for the Heilongjiang Provincial Universities, Scientific Research Fund of Harbin Medical University-Daqing (2018XN-23).

## Conflict of Interest

The authors declare that the research was conducted in the absence of any commercial or financial relationships that could be construed as a potential conflict of interest.

## Publisher's Note

All claims expressed in this article are solely those of the authors and do not necessarily represent those of their affiliated organizations, or those of the publisher, the editors and the reviewers. Any product that may be evaluated in this article, or claim that may be made by its manufacturer, is not guaranteed or endorsed by the publisher.
