# Supplementary material for: Potential Association Between Changes in Microbiota Level and Lung Diseases: A Meta-Analysis
Source: Front Med (Lausanne). 2022 Jan 14;8:723635. doi: 10.3389/fmed.2021.723635 (PMC8795898; doi:10.3389/fmed.2021.723635)
Supplement: Supplementary file 1 [file Table_1.DOCX]

Supplementary Material

# Supplementary Figures

**Supplementary Figure 1. Forest plots of the association between *A. baumannii*, *E. cloacae*, *P. aeruginosa*, *S. aureus*, *A. baumannii*  level and asthma patients.**

**Supplementary Figure 2. Forest plots of the association between *A. baumannii*, *E. cloacae*, *P. aeruginosa*, *S. aureus*, *A. baumannii*  level and COPD patients.**

**Supplementary Figure 3.**Meta-analysis of influence analysis.

**Supplementary Figure 4.** Meta-analysis of publication bias analysis.

**Searched Strategy**

**Pubmed**

Search: (((((((Chronic Obstructive Pulmonary Diseases[Title/Abstract]) OR (COPD[Title/Abstract])) OR (Idiopathic Pulmonary Fibrosis[Title/Abstract])) OR (Asthma*[Title/Abstract])) OR (Pneumonia[Title/Abstract])) OR ((((("Lung Diseases"[Mesh]) OR "Asthma"[Mesh]) OR "Pulmonary Disease, Chronic Obstructive"[Mesh]) OR "Pneumonia"[Mesh]) AND "Lung Diseases, Fungal"[Mesh])) AND ((((Microbiota*[Title/Abstract]) OR (Microbial Communit*[Title/Abstract])) OR (Microbiome*[Title/Abstract])) OR (("Microbiota"[Mesh])))) AND (("2010/01/01"[Date - Publication] : "2021/03/20"[Date - Publication])) Sort by: Most Recent

**Embase**

lung AND microbiota*:ab,ti OR 'microbial communit*':ab,ti OR microbiome*:ab,ti

'lung microbiota'/exp OR 'microbiome'/exp OR 'microbial community'/exp

'lung disease'/exp OR 'chronic obstructive lung disease'/exp OR 'fibrosing alveolitis'/exp OR 'asthma'/exp OR 'pneumonia'/exp

'lung disease':ab,ti OR 'chronic obstructive lung disease':ab,ti OR 'idiopathic pulmonary fibrosis':ab,ti OR ' asthma':ab,ti

**Web of science**

**Lung Diseases** (Topic) or **Chronic Obstructive Pulmonary Diseases** (Topic) or **Idiopathic Pulmonary Fibrosis** (Topic) or **COPD** (Topic) or **Asthma** (Topic) or **Pneumonia** (Topic)

**Lung microbiota** (Topic) or **lung microbiome** (Topic) or **microbial community** (Topic)

**PY= (2010-2021)**
